# First Case of Candidemia With Fluconazole‐Resistant *Candida albicans* and Y132H Mutations in *ERG11* Gene From Pakistan

**DOI:** 10.1155/crdi/2911301

**Published:** 2026-08-13

**Authors:** Nosheen Nasir, Joveria Farooqi, Iffat Khanum, Sadaf Zaka, Shariqa Batool, Najia Ghanchi, Kauser Jabeen

**Affiliations:** ^1^ Department of Medicine, Aga Khan University, Karachi, Pakistan, aku.edu; ^2^ Department of Pathology and Laboratory Medicine, Aga Khan University, Karachi, Pakistan, aku.edu; ^3^ Medical College, Aga Khan University, Karachi, Pakistan, aku.edu

**Keywords:** *ERG11* gene, fluconazole-resistant *Candida albicans*, Y132H mutation

## Abstract

*Candida albicans* is one of the critical priority fungal pathogens as classified by the World Health Organization. Fluconazole resistance in *C. albicans* is increasingly being reported from different parts of the world. The epidemiology of antifungal resistance in Pakistan is evolving. While fluconazole resistance is rarely reported in invasive candidemia, it has been identified in noninvasive *Candida* isolates. Here, we describe the first case of candidemia with fluconazole‐resistant *C*. *albicans* with Y132H mutations in the *ERG11* gene from Pakistan. A 36‐year‐old male patient with comorbid conditions, including diabetes mellitus, hypertension, and a recent history of critical COVID‐19 pneumonia, presented to the hospital emergency department with septic shock secondary to Fournier’s gangrene. Blood culture confirmed fluconazole‐resistant *C. albicans*. Our case report underscores the urgent need for antifungal stewardship to prevent the escalating issue of antifungal resistance.

## 1. Introduction

Antifungal resistance is an emerging global threat with significant implications for patient outcomes and the cost of care. The problem is further amplified in low‐ and middle‐income countries where diagnostic capacity and treatment options are already limited, making these infections difficult to manage. *Candida albicans* is one of the critical priority fungal pathogens classified by the World Health Organization (WHO) [1]. Fluconazole resistance in *C. albicans* is increasingly being reported from different parts of the world. A recent meta‐analysis evaluating resistance in *C. albicans* isolated from oral samples has reported a resistance rate of 2.1% in 5539 isolates, with significantly higher resistance rates in patients with HIV/AIDS and cancers [2]. Rates of fluconazole‐resistant *C. albicans* in candidemia cases are comparatively lower [2, 3]. Reports of fluconazole resistance in *C. albicans* from Pakistan are scarce and reported mainly in noninvasive isolates [4–7]. This may be attributable to differences in testing methodologies and types of specimens tested. Here, we describe a patient who was hospitalized with fluconazole‐resistant candidemia secondary to Fournier’s gangrene.

## 2. Case Presentation

A 36‐year‐old male, known case of diabetes mellitus and hypertension, presented to the hospital emergency department with septic shock secondary to Fournier’s gangrene in April 2022. In January 2022, he was admitted to the hospital with critical COVID‐19 pneumonia. The patient had a history of chronic backache and long‐term intravenous nalbuphine and dexamethasone use. He also had excessive alcohol intake and had iatrogenic Cushing’s syndrome. Magnetic resonance imaging (MRI) of the lumbar spine showed multiple osteoporotic fractures, likely secondary to chronic steroid use. He had not fully recovered from critical COVID‐19, for which he had required a 13‐day hospitalization with noninvasive ventilation and systemic steroids.

**FIGURE 1:**
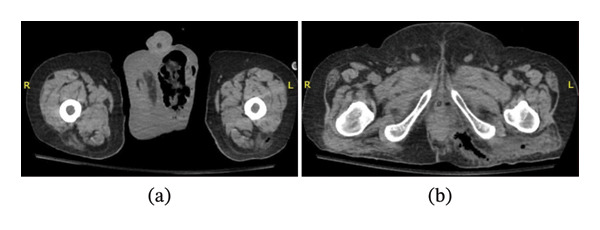
(a, b) Multiple soft tissue gas locules seen within the left hemiscrotum extending into the ischiorectal fossa, left inguinal region, and left anterior abdominal wall suggestive of Fournier’s gangrene.

**FIGURE 2:**
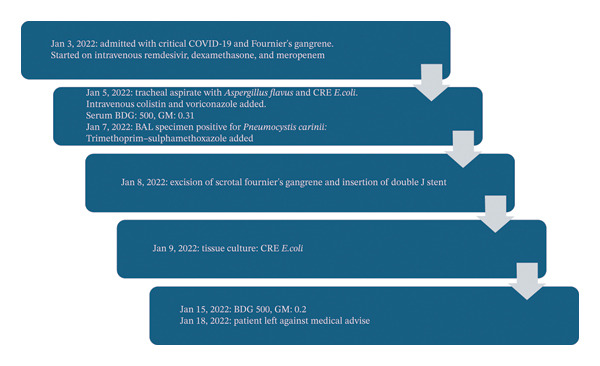
Timeline of events during hospitalization in January 2022.

During the same admission in January 2022, he was diagnosed with Fournier’s gangrene when he complained of bilateral scrotal swelling and scrotal pain for 3 days before presentation. Pain was of moderate intensity and pricking in character with radiation to the bilateral flank regions. Scrotal ultrasound showed diffuse thickening and edema of the scrotal wall with interspersed multiple echogenic gas foci in the scrotum, suggestive of Fournier’s gangrene, but testes and epididymides were spared. A computed tomography scan of the kidneys, ureters, and bladder (CT KUB) was also performed, which showed a nonobstructing right proximal ureteric calculus and soft tissue gas locules in the left hemiscrotum extending into the ischiorectal fossa, left inguinal region, and anterior abdominal wall, suggestive of Fournier’s gangrene (Figures 1a and b). He underwent right double J stenting along with bladder biopsy and debridement of Fournier’s gangrene as well as suprapubic catheter placement and was empirically started on intravenous meropenem. Tissue culture grew carbapenem‐resistant *E. coli* sensitive to fosfomycin. A peripherally inserted central catheter (PICC) line was inserted for administration of intravenous meropenem and fosfomycin. Due to persistent shortness of breath and worsening chest infiltrates on chest radiograph, a tracheal aspirate was sent, which grew *Aspergillus flavus* complex, with serum beta‐D‐glucan level of 500 pg/mL (Fungitell, Cape Cod Inc., USA). Oral voriconazole was started, suspecting invasive aspergillosis despite serum galactomannan index of 0.3 (PlateliaBio‐Rad, USA). Two days later, *Pneumocystis jirovecii* DNA was detected in bronchoalveolar lavage fluid using a nucleic acid amplification assay (RealStar assay, Altona Diagnostics, Hamburg, Germany). Patient required further medical care as well as surgical debridement for Fournier’s gangrene but left against medical advice to pursue treatment at another hospital on 18^th^ January 2022 (Figure 2).

In April 2022, the patient developed hospital‐acquired pneumonia while receiving treatment at another hospital. His condition worsened, requiring invasive mechanical ventilation, and he was referred to our hospital for further management. On arrival at the ER, he was in septic shock with disseminated intravascular coagulopathy (DIC). He was admitted to the intensive care unit (ICU) and required mechanical ventilation and vasopressor support. As the Fournier’s gangrene had progressed and a rectocutaneous fistula had developed, he underwent emergency orchidectomy.

Empirical treatment with intravenous meropenem, vancomycin, and colistin was started. Two sets of blood culture (BacT/Alert, BioMérieux, France) sent at the time of admission grew fluconazole‐resistant *C albicans* after 3 days of incubation. The organism was identified by green colonies on chromogenic medium (BD BBL CHROMagar *Candida*, BD Life Sciences, USA), cycloheximide tolerance, positive germ tube test, and excellent identity on VITEK2 Yeast ID card (BioMérieux, France). Antifungal susceptibility (AFST) was performed using YeastOne Sensititre YO10 (Trek Diagnostics, Denmark) following the methodology as recommended by the manufacturer and interpreted as per CLSI M60Ed2 [8]: categorizing fluconazole susceptible at MIC ≤ 2 μg/mL, susceptible dose‐dependent at 4 μg/mL, and resistant at ≥ 8 μg/mL; voriconazole susceptible at MIC ≤ 0.12 μg/mL, intermediate at 0.25–0.5 μg/mL, and resistant at ≥ 1 μg/mL; and caspofungin susceptible at MIC ≤ 0.25 μg/mL, intermediate at 0.5 μg/mL, and resistant at ≥ 1 μg/mL. The quality control strains used for AFST testing were ATCC 6258 *Pichia kudriavzevii* (*Candida krusei*) and ATCC 22019 *Candida parapsilosis*.

Given the patient’s risk factors for invasive candidiasis and sepsis, *C albicans* growth was considered significant. The likely source was Fournier’s gangrene since tissue culture also grew *C*. *albicans,* which also underwent AFST. Treatment was started with intravenous amphotericin B deoxycholate at a dose of 0.7 mg/kg/day. Caspofungin and liposomal amphotericin could not be started due to high cost and inconsistent availability in Pakistan at the time. Fluconazole MIC came out to be 8 μg/mL, while voriconazole MIC was 0.5 μg/mL. Hence, fluconazole was interpreted as resistant, voriconazole was interpreted as intermediately susceptible, while caspofungin and amphotericin were sensitive at MICs of 0.015 and 0.25 μg/mL, respectively. Subsequently, 2 additional sets of blood cultures sent after 48 h also turned positive for the same organism [8]. The *ERG11* gene region of 1587 bp was amplified, and PCR products were sequenced using Sanger sequencing methodology (Eurofins USA). The sequences were mapped against available *ERG11* sequences from gene bank compared to X13296.1, using Molecular Evolutionary Genetics Analysis 11 (MEGA11) software. Two nonsynonymous mutations, Y132H and T123I, and one synonymous mutation, T909A, were found.

**FIGURE 3:**
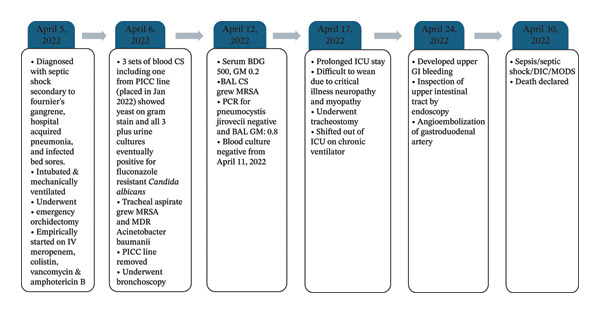
Timeline of events during hospitalization in April 2022.

The patient’s hospital course was complicated by acute kidney injury secondary to sepsis‐induced acute tubular necrosis and amphotericin‐induced nephrotoxicity. Blood cultures were repeated after 5 days and were reported negative after 120 h of incubation. He developed critical illness myopathy and neuropathy and required invasive mechanical ventilation. He developed upper gastrointestinal bleeding and required multiple blood product transfusions and underwent urgent angioembolization of the gastroduodenal artery. Despite all efforts, his condition kept deteriorating, and he passed away after a month of hospitalization (Figure 3).

## 3. Discussion

We describe a patient with multiple comorbid conditions and risk factors for invasive fungal infection who developed fluconazole‐resistant candidemia secondary to Fournier’s gangrene, and despite intervention, had an adverse outcome.

*C. albicans* is one of the most virulent *Candida* species. Emergence of resistance in a virulent species that is also difficult to treat can be a serious problem. Risk factors of invasive candidiasis are quite well studied and include hematologic or solid organ malignancies, prolonged hospitalization, especially in ICUs, surgery, use of immunosuppressants, antibiotics, and devices [9]. Risk factors for resistant invasive candidiasis include prior exposure to azoles [10]. Our patient had several risk factors, including invasive procedures and lines, prior prolonged steroid use, intensive care, and prior use of voriconazole.

Amphotericin B deoxycholate was used for treatment. Both IDSA and ECMM candidemia guidelines recommend an echinocandin as first‐line therapy [11, 12]. In our setting, due to cost and access constraints to echinocandins and liposomal amphotericin B, use of amphotericin B deoxycholate is common. Therefore, rigorous monitoring for amphotericin‐related adverse effects is required.

In this study, MICs for *C*. *albicans* were determined using the Sensititre YeastOne colorimetric broth microdilution (BMD) platform. Comparative evaluations have demonstrated that Sensititre YeastOne performs closely to the CLSI reference BMD (M27) method for susceptibility testing of *Candida* species with overall > 90% essential agreement and > 90% categorical agreement for azoles, echinocandins, and amphotericin B [13–15]. Although some studies have shown a lower essential and categorical agreement for azoles [13, 16], however, the majority of data support the reliability of Sensititre YeastOne in routine clinical practice. We acknowledge our limitation in not confirming MICs using reference BMD, as actual MIC values may vary between the two methodologies.

Fluconazole is an inhibitor of the Cytochrome P450 enzyme lanosterol demethylase in the ergosterol biosynthesis pathway encoded by the *ERG11* gene. *ERG11* gene mutations reduce drug binding, resulting in arrested cell growth due to the accumulation of methylated sterols in cell membranes of fungal species [17]. With most substitutions related to the azole‐resistant species, they may either modify the predicted catalytic enzyme site or a heme interaction surface site or may sometimes affect the fungal‐specific external loop. In addition, some single mutations, such as substitution of single amino acids such as Y132F, are notorious for conferring fluconazole resistance in *C. tropicalis*, *C. auris*, and *C. parapsilosis* [18]. Other point mutations may involve deletion mutations with truncation of Erg11p; a missense mutation with minimal ergosterol production; or specific point mutations such as Y132F, K143R, and F126T, exhibiting elevated MIC upon treatment with fluconazole [19].

The mutation Y132F in *ERG11* is known to reduce susceptibility to fluconazole in *C. auris* and *C. parapsilosis* from Pakistan. A study revealed a nonsynonymous mutation, Y132F, in the *ERG11* gene in about 86% of fluconazole‐resistant isolates, which are usually resistant to all triazoles [20]. Another nonsynonymous mutation, T123I, was also detected in the *ERG11* gene. Less often reported than Y132H, this mutation has been observed in azole‐resistant *C. albicans* isolates [21]. The synergistic impact of Y132F and T123I plays a role in reducing azole susceptibility. T909A, a synonymous mutation, was also reported. However, it is unlikely to impact drug susceptibility but may serve as an epidemiological marker. *C. albicans* is one of the most virulent *Candida* species, much more so than *C. parapsilosis*. Emergence of resistance in a virulent species that is also difficult to treat can be a serious problem [20, 22].

Apart from fluconazole, the isolate also had reduced susceptibility to voriconazole, indicating azole‐class resistance. Prior exposure to voriconazole is well known to exert class‐level selective pressure, promoting the emergence of *ERG11* point mutations that reduce azole binding affinity and contribute to elevated fluconazole and voriconazole MICs [23]. These molecular mechanisms provide a biologically plausible explanation linking prior azole exposure with the patient’s observed resistance phenotype.

## 4. Conclusion

Our case illustrates the clinical and therapeutic challenges posed by resistant invasive *Candida albicans* and highlights the need for strengthening antifungal stewardship and establishing coordinated surveillance systems to guide evidence‐based therapy, monitor emerging resistance patterns, and improve outcomes in patients with invasive candidiasis.

## Author Contributions

All authors contributed to the manuscript.

## Funding

No specific funding has been used for drafting this manuscript.

## Disclosure

All the authors have read and approved the final manuscript.

## Ethics Statement

This case report was conducted in accordance with institutional ethical standards and the principles of the Declaration of Helsinki. Ethical approval was obtained from the Aga Khan University Ethics Review Committee (ERC Reference number: 2022‐7706‐21915). The requirement of written informed consent was waived due to the retrospective nature of the study and use of de‐identified information.

## Consent

Please see the Ethics Statement. All patient data in this case report have been thoroughly anonymized, and personally identifiable information has been removed or altered to protect the patient’s privacy.

## Conflicts of Interest

The authors declare no conflicts of interest.

## Supporting Information

Additional supporting information can be found online in the Supporting Information section.

## Supporting information


**Supporting Information** CARE‐checklist‐English‐2013_1.

## Data Availability

Data are available on request due to privacy/ethical restrictions.
